# Gastroprotective Effect of Polypeptide-K Isolated from *Momordica charantia*'s Seeds on Multiple Experimental Gastric Ulcer Models in Rats

**DOI:** 10.1155/2022/6098929

**Published:** 2022-01-12

**Authors:** Nurul 'Ain Abu Bakar, Muhammad Nazrul Hakim Abdullah, Vuanghao Lim, Yoke Keong Yong

**Affiliations:** ^1^Department of Biomedical Science, Faculty of Medicine and Health Sciences, Universiti Putra Malaysia, 43400 UPM Serdang, Selangor, Malaysia; ^2^Department of Medical Science and Technology, Faculty of Health Sciences, PICOMS International University College, 68100 Kuala Lumpur, Malaysia; ^3^Advanced Medical and Dental Institute, Universiti Sains Malaysia, Bertam 13200, Kepala Batas, Penang, Malaysia; ^4^Department of Human Anatomy, Faculty of Medicine and Health Sciences, Universiti Putra Malaysia, 43400 UPM Serdang, Selangor, Malaysia

## Abstract

Peptic ulcer disease is a multifactorial disorder and is the most significant public health concern nowadays. Previous study showed that essential oil extracted from *Momordica charantia*'s seed exhibited gastroprotective effect. However, the evidence for the gastroprotective effect of its active compound, polypeptide K (PPK), remains unclear. This study aimed to examine the preventive effect of PPK against different experimental gastric lesions models in rats. The possible gastroprotective effect of PPK was assessed in hydrochloride ethanol- and indomethacin-induced gastric ulcer models in *Sprague Dawley* rats and was further evaluated macroscopically and microscopically. Pyloric ligation experiments were used to investigate gastric secretion. Oral administration of PPK at all concentrations (10, 25, and 50 mg/kg) showed significant (*p* < 0.05) reduction in total area of lesion in both hydrochloride ethanol- and indomethacin-induced gastric ulcer models. The highest inhibition rate was seen in PPK dose of 50 mg/kg with 64.9% and 72.2% on hydrochloride ethanol and indomethacin models, respectively. Microscopically, PPK preserved the normal architectures of the gastric tissues from being damaged by hydrochloride ethanol and indomethacin. Further, in the pyloric ligation studies, PPK significantly (*p* < 0.05) decreased the ulcer area where the highest protection was exhibited by 50 mg/kg with 70% inhibition rate. Moreover, all concentrations of PPK also significantly (*p* < 0.05) enhanced the gastric wall mucus secretion. Collectively, this study demonstrated the gastroprotective effect of PPK on hydrochloride ethanol- and indomethacin-induced gastric ulcer models. The possible mechanism might be associated with enhanced mucus secretion and thus lowering the total acidity.

## 1. Introduction

Despite significant progress in our knowledge of peptic ulcer disease, its etiology still remains largely unknown. The increasing usage of nonsteroidal anti-inflammatory drugs (NSAID) is one of the most well-known causal factors for peptide ulcer disease [1]. Other than that, oxidative stress, alcohol consumption, smoking, *Helicobacter pylori* infection, and imbalance between exogenous damaging agents and endogenous protective mechanisms also result in gastrointestinal bleeding and ulceration [2]. It is one of the dangerous gastrointestinal diseases in the world, affecting roughly 5–10% of the global population [3], with a substantial influence on morbidity and mortality globally, posing a huge burden on healthcare resources. There is a need to identify potential alternatives particularly from natural compounds that might be a viable replacement for current treatment regimens to this global challenge due to the increasing incidence of gastric ulcers. In addition, natural compounds from plant usually have less adverse effect compared with some of the synthetic medications used to treat or manage the disease.

Previous study documented that essential oil extracted from *Momordica charantia*'s seeds possessed gastroprotective properties against hydrochloride/ethanol and indomethacin [4]. *Momordica charantia* L. (MC) from Cucurbitaceae family is usually called bitter melon or bitter gourd that is native to Thailand, India, and Africa's semitropical climates. It is consumed as a vegetable in many countries, but it has often been used in a number of Asian traditional medicine systems to treat a variety of illnesses since ancient times, including ulcer [5], inflammation [6], and hyperglycemia [7]. The seed is particularly used in the treatment of diabetes, stomachache, intestinal parasites, intestinal gas, ulcer, and liver and spleen problems [8] traditionally, while pharmacological actions that have been documented match the traditional usage, such as antihyperglycemic [9], antiulcerogenic [4], and hepatoprotective effects [10].

Polypeptide K (PPK), an active protein isolated from the seeds of MC, has been shown to have antidiabetic effect [9, 11] which is more effective than that of polypeptide P, another active protein from the seeds of MC extracted by acid ethanol extraction [9]. In addition, Garg and his research team also documented that PPK significantly inhibits the enzyme level of *α*-glucosidase and *α*-amylase up to 79.18% and 35.58% level [12] which is important in the regulation of blood glucose. Hence, the current study aimed to determine the antiulcerogenic effect of PPK against hydrochloride/ethanol-, indomethacin-, and pyloric ligation-induced ulceration in stomach of an animal model.

## 2. Materials and Methods

### 2.1. Chemicals and Reagents

Polypeptide K (PPK) (batches 37/11, 121/11, and 375/11) was graciously given by Magna Bio-Laboratories Sdn. Bhd. Malaysia. Kanna's technique was used to separate polypeptide K from its precursor [13]. Alcian blue, diethyl ether, absolute ethanol, and paraffin were from Merck, Germany; hematoxylin and eosin were from Sigma Chemicals (St. Louis, MO, USA); and xylene was from BDH Chemicals, UK.

### 2.2. Experimental Animals and Ethic Statement

Sprague Dawley male rats, weighing 150 g–200 g, were used to evaluate the gastroprotective effect of PPK. The experimental animals were housed in pairs in conventional cages and were kept under standard conditions of 12-hour light/dark cycles at a constant temperature of 25 ± 3°C. The animals were given access to regular laboratory animal diet and water on an ad libitum basis. The studies were conducted in compliance with the standards and were authorized by the Faculty of Medicine and Health Sciences, Universiti Putra Malaysia's Institutional Animal Care and Use Committee (IACUC) (UPM/FPSK/PADS/BR-UUH/00275). Prior to the trial, all animals were acclimated to the laboratory environment for a minimum of three days.

### 2.3. Grouping and Dosing of Animals

The rats were randomly allocated to six groups of six rats each (*n* = 6). An oral gavage was used to administer all of the treatments once a day for seven days. The gastric ulcer was induced on day 7 after being administrated with the test compound for 1 hour.

#### 2.3.1. Hydrochloride Ethanol (HCl/EtOH) Induced Ulcer Model

- Group 1: distilled water
- Group 2: distilled water + HCl/EtOH
- Group 3: ranitidine (100 mg/kg) + HCl/EtOH
- Group 4: 10 mg/kg PPK + HCl/EtOH
- Group 5: 25 mg/kg PPK + HCl/EtOH
- Group 6: 50 mg/kg PPK + HCl/EtOH

Briefly, gastric ulcer was induced on day 7 after being pretreated with testing compound for 1 hour by oral administration of 1 mL HCl/EtOH solution (1 mL/60% EtOH containing 150 mM HCl) [4]. Animals were euthanized 3 hours after induction of ulcer under carbon dioxide chamber on the 7th day. The stomach was excised and cleaned with normal saline. Then, the stomach was next dissected to check for hemorrhagic lesions and severity of ulcers microscopically. Ulcer index was calculated using scoring system adapted from Miñano et al. [14].

#### 2.3.2. Indomethacin-Induced Ulcer Model

- Group 1: distilled water
- Group 2: distilled water + indomethacin
- Group 3: ranitidine (100 mg/kg) + indomethacin
- Group 4: 10 mg/kg PPK + indomethacin
- Group 5: 25 mg/kg PPK + indomethacin
- Group 6: 50 mg/kg PPK + indomethacin

Briefly, the rats that fasted for 24 hours were induced with gastric ulcer by administrating 1 mL of indomethacin solution (1 mL, 100 mg/kg, dissolved in distilled water) 1 hour after pretreatment on day 7 [15]. The rats were sacrificed under carbon dioxide chamber and the stomachs were removed after 6 hours of induction. All the stomachs were examined macroscopically and microscopically for the calculation of ulcer lesion and ulcer index according to the scoring system as mentioned previously [14].

### 2.4. Evaluation of Histopathological Changes of Stomach Tissues

Stomach tissues were taken from the previous experiment and fixed in 10% neutral buffered formalin for 24 hours before preparing paraffin-embedded sections (5M). After staining with hematoxylin and eosin (H&E), the sections were mounted with DPX mounting solution. To assess the alterations in histomorphology, selected sections were examined under a light microscope. The degree of ulceration severity was determined using a validated grading method developed from Farrell et al. [16]. After reviewing all slides for inflammatory spectrum, the inflammatory scoring system was devised, and the following scores were assigned:

- Normal (0)
- Inflammatory cells at a minimum (1)
- Inflammatory cells in a moderate quantity (2)
- Inflammatory cells in large numbers (3)

### 2.5. Pyloric Ligation-Induced Ulcer Model

The experiment methodology was modified from Shay et al. [17]. All the animals were fasted for 48 hours but allowed free access to water. All treatments were made 1 hour prior to pylorus ligation. Pylorus ligation was done on all of the rats in the preceding groups, while they were under moderate anaesthesia with ketamine. During the postoperative phase, the animals were given water but not fed. After 6-hour ligation, the animals were promptly killed through carbon dioxide overdose and decapitated and then their stomachs taken. Gastric juice was collected in clean centrifuge tubes after the stomach was sliced along its greater curvature. After centrifuging the contents at 2000 rpm for 10 minutes, the gastric juice was measured. Supernatant pH and total acidity were also measured.

### 2.6. Measurement of Gastric Acidity

Briefly, 1 mL of gastric juice was tested for acidity by titrating with 0.01 N NaOH until a persistent pink color was seen. The total acidity was expressed as mEq/L.

### 2.7. Calculation of the Mucus Content of the Stomach Wall

Gastric wall mucus was analyzed by using modified procedure by Piper et al. [18]. The stomach was opened along its great curvature before being washed in cold saline. The glandular portion of the stomach was removed, weighed, and rinsed with 0.16 M sucrose solution buffered. Then, the stomachs were stained in 10 mL 0.1% w/v Alcian blue for 2 hours. The excess dye was removed with 0.25 M sucrose, and the mucus-bound dye complex was recovered by immersing the stomach tissue in a 0.5 M MgCl_2_ solution for 1 minute at 30-minute intervals for 2 hours. The aqueous phase was quantitated at OD 580 nm.

### 2.8. Statistical Analysis

All data were presented as the mean ± standard error of mean (SEM) and were analyzed by one-way ANOVA followed by Dunnett test using SPSS software, where *p* < 0.05 was considered to be significant.

## 3. Results

### 3.1. PPK Inhibited HCl/EtOH-Induced Gastric Ulcer Macroscopically

**Figure 1:**
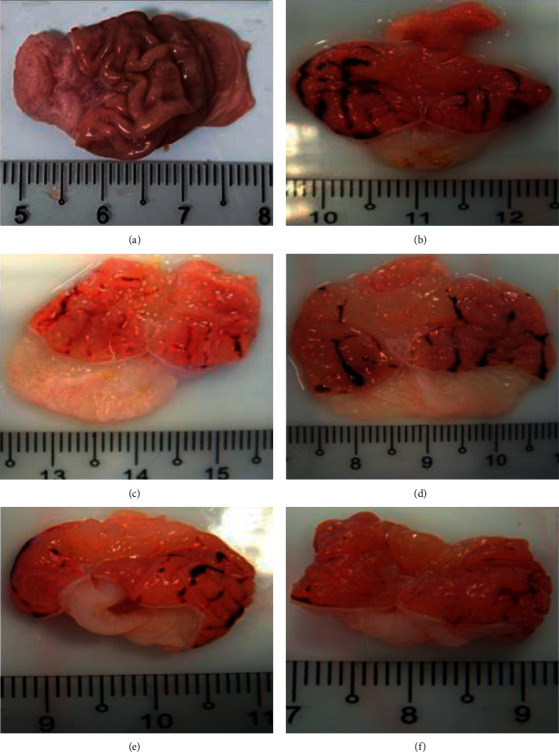
Representative rat's stomach photomicrographs that were cut along the greater curvature. (a) Control, (b) HCl/EtOH, (c) reference drug (ranitidine), (d) PPK 10 mg/kg, (e) PPK 25 mg/kg, and (f) PPK 50 mg/kg, indicating the presence of many circular and linear stomach ulcers in the HCl/EtOH group, which were considerably decreased by ranitidine and PPK.

HCl/EtOH-induced severe mucosal injury in the rats without any treatment with total area of gastric lesion was 44.8 ± 3.5 mm^2^ (Figure 1) as compared to the rats in control group (without gastric ulcer induction). The isolated gastric taken from HCl/EtOH treated rats showed many circular and longitudinal ulcers. Interestingly, the rats pretreated with PPK at the dosage of 50 mg/kg significantly reduced total area of gastric lesion, which was 15.67 ± 2.67 mm^2^ as compared to HCl/EtOH group (Table 1). This showed 64.9% protection by 50 mg/kg of PPK against HCl/EtOH-induced gastric ulceration. On the other hand, both 10 and 25 mg/kg of PPK reduced the ulcer index (35.67 ± 3.84 mm^2^ and 33.16 ± 4.13 mm^2^, respectively) but somehow there was no significant difference compared to the HCl/EtOH group; meanwhile reference drug, ranitidine, significantly reduced the gastric index. This data indicated that only the high dosage of PPK was able to suppress HCl/EtOH-induced gastric ulceration.

### 3.2. PPK Inhibited HCl/EtOH-Induced Gastric Ulcer Microscopically

**Figure 2:**
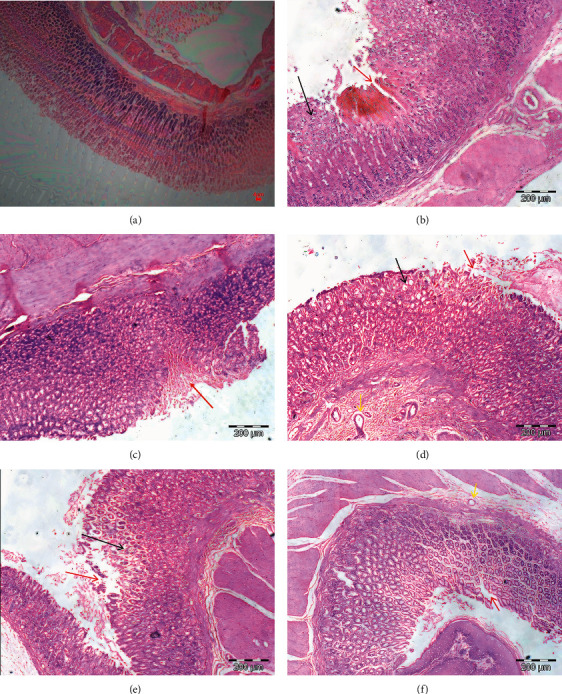
Effect of PPK on histopathological changes of the stomach of rats with HCl/EtOH-induced gastritis. (a) Control, (b) HCl/EtOH, (c) reference drug (ranitidine), (d) PPK 10 mg/kg, (e) PPK 25 mg/kg, and (f) PPK 50 mg/kg. The alterations in stomach tissue slide section were confirmed by staining with H&E and then observed at a magnification of 100x.

Figure 2(a) illustrates a section from an animal that received only distilled water and was not subjected to ulcer induction. Gastric mucosal layer histomorphology revealed normal tissue architecture and the absence of gastric tissue degeneration. Meanwhile the histopathological examination of the stomachs from the groups that were administrated with HCl/EtOH caused remarkable gastric injury, as evidenced by the existence of edema and/or vacuolation, epithelial disruption, and extensive erosion of the muscularis mucosa (Figures 2(b)–2(f)). Animals pretreated with 25 and 50 mg/kg of PPK and ranitidine had markedly increased mean score of normal features compared with HCl/EtOH alone group (Table 2). On top of that, 50 mg/kg of PPK and ranitidine also significantly reduced epithelial disruption mean score, 1.50 ± 0.67 for each group, respectively, compared with HCl/EtOH group (5.00 ± 0.63). None of the treatment groups, except ranitidine, successfully reduced the edema/vacuolation, and all treatments failed to suppress HCl/EtOH-induced erosion extending to the muscularis mucosae.

### 3.3. PPK Suppressed Indomethacin-Induced Gastric Ulcer Macroscopically

**Figure 3:**
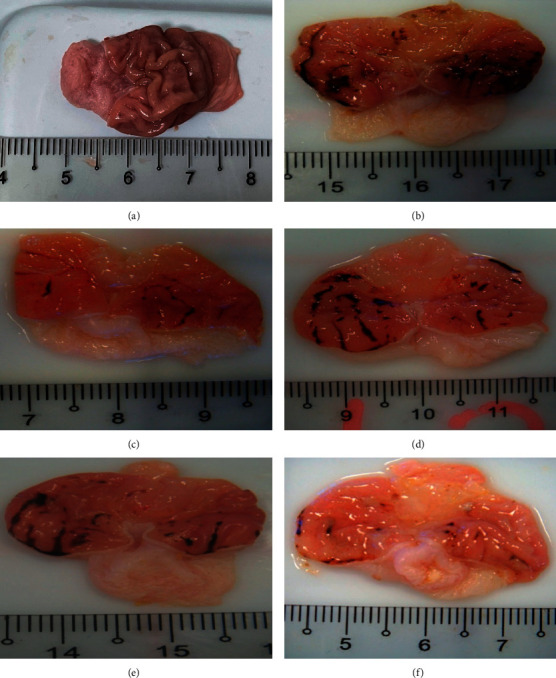
Representative rat's stomach photomicrographs that were cut along the greater curvature. (a) Control, (b) indomethacin, (c) reference drug (ranitidine), (d) PPK 10 mg/kg, (e) PPK 25 mg/kg, and (f) PPK 50 mg/kg, indicating the presence of many circular and linear stomach ulcers in the indomethacin group, which were considerably decreased by ranitidine and PPK.

Table 3 and Figure 3 show that pretreatment of PPK in all concentrations significantly suppressed gastric ulcer induced by indomethacin by reducing the total area of lesions in a dose-dependent manner. PPK at the dosages of 10, 25, and 50 mg/kg significantly (*p* < 0.05) reduced the total area of gastric lesion from 41.33 ± 3.57 to 23.00 ± 3.38, 17.33 ± 3.56, and 11.50 ± 2.01, respectively (Table 3). On top of that, reference drug ranitidine also significantly reduced the total area of lesion up to 67% compared to HCl/EtOH alone group.

### 3.4. PPK Suppressed Indomethacin-Induced Gastric Ulcer Microscopically

**Figure 4:**
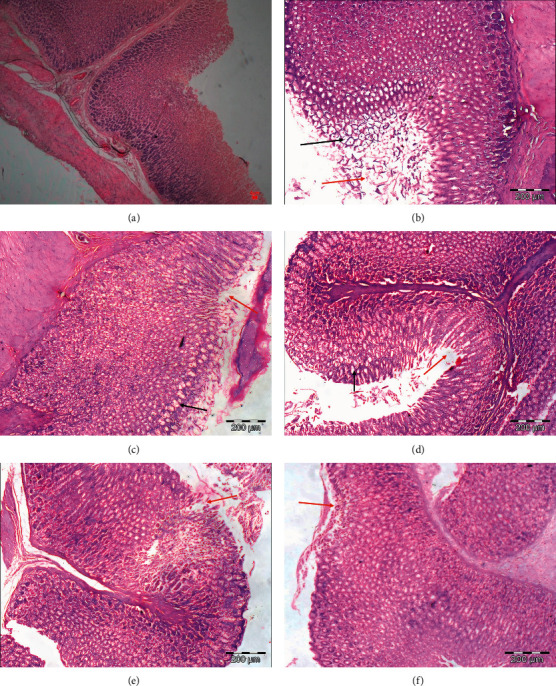
Effect of PPK on histopathological changes of the stomach of rats with indomethacin-induced gastritis. (a) Control, (b) HCl/EtOH, (c) reference drug (ranitidine), (d) PPK 10 mg/kg, (e) PPK 25 mg/kg, and (f) PPK 50 mg/kg. The alterations in stomach tissue slide section were confirmed by staining with H&E and then observed at a magnification of 100x.

Administration of indomethacin caused injury to the gastric mucosa with increasing the mean score of the pathological features (Table 4). PPK at 10 and 25 mg/kg failed to suppress edema or vacuolation, epithelial disruption, and erosion that has progressed to the muscularis mucosae (Table 4, Figure 4) against indomethacin-induced gastritis. Moreover, both concentrations also were not able to increase the normal features of the stomach tissues. However, rats pretreated with 50 mg/kg of PPK significantly (*p* < 0.05) decreased the whole damage induced by indomethacin compared to indomethacin alone group (Table 3). Similarly, ranitidine significantly decreased edema and/or vacuolation and epithelial disruption, while it increased normal features of the gastric tissue against the indomethacin.

### 3.5. PPK Decreased Pathological Changes in Pylorus-Ligated Rats

As demonstrated in Table 5, both PPK and the reference drug, ranitidine, considerably decreased ulceration area, with 50 mg/kg of PPK exhibiting the greatest protection rate (70% inhibition rate), while only ranitidine and the highest concentration of PPK were able to reduce the total acidity compared to disease model. However, no significant changes were observed as compared to the indomethacin group in the amount and pH of gastric juice for any of the treatment groups. However, all concentrations of PPK and reference drug significantly increased mucus secretion from gastric wall when compared to indomethacin group (0.89 + 0.05 Alcian blue mg/g wet tissue), where the highest secretion was produced by 50 mg/kg of PPK, 2.60 + 0.04 Alcian blue mg/g wet tissue (Table 6).

## 4. Discussion

A number of factors induce gastric ulcer formation; however, it is widely acknowledged that it is caused by an imbalance between aggressive factors and endogenous defense mechanisms. Diverse treatments and medications are employed to restore the equilibrium between damaging and protecting factors, including inhibiting gastric acid release, increasing mucosal output, stabilizing surface epithelial cells, and disrupting the synthesis of prostaglandin [19]. Unfortunately, no drug has met all the goals of gastric ulcer therapy till date. Apart from this, patients who suffer from the gastric ulcer have reported tolerating traditional antiulcer medications. Also, long-term use of these medications has substantial side effects, thus necessitating the development of new and safer antiulcer therapies.

Our previous study showed that essential oil extracted from the *Momordica charantia* (MC) seeds exhibited antiulcer properties against hydrochloride ethanol (HCl/EtOH), indomethacin, and pylorus ligation [4]. This proved that MC seed contains antiulcerogenic bioactive compound. Polypeptide K (PPK), a polypeptide isolated from the MC seed, is well known for its antidiabetic properties [9, 12, 20]. To our knowledge, no comprehensive investigation has been done on PPK's antiulcer properties. The current investigation was conducted to assess polypeptide K's antiulcerogenic activity in three distinct animal models: hydrochloride ethanol- (HCl/EtOH-), indomethacin-, and pylorus ligation-induced ulceration formation in vivo.

Numerous earlier researches examined the pathophysiology of hydrochloride ethanol (HCl/EtOH) in the development of gastric ulcers. The combination of hydrochloride and ethanol causes significant damage to the stomach mucosa; for example, ethanol causes necrotizing lesions through direct necrotizing action, lowering defense systems such as bicarbonate secretion and mucus production [21]. In addition, generation of free radicals, leukotriene C4, and mast cell secretory products also strongly associated with the ethanol-induced gastric ulcer [22]. Addition of hydrochloride acid (HCl) to the treatment causes a fast and severe stomach damage [21]. Therefore, HCl/EtOH-induced gastric ulcers are regarded as a valid method for assessing the effectiveness of gastroprotectants. Pretreatment with PPK at a concentration of 50 mg/kg markedly decreased the total lesion area from 44.67 ± 3.45 mm^2^ to 15.67 ± 2.67 mm^2^ compared to disease group, by giving 65% of protection in rats treated with HCl/EtOH. This result showed bigger effect compared to reference drug, ranitidine, which gave around 32% of protection rate. On the other hand, a dose of 50 mg/kg also significantly suppressed epithelial disruption and enhanced the normal features of the gastric wall tissue in the rats treated with the HCl/EtOH; on a microscopic level, its gastroprotective action is equivalent to that of ranitidine. These data show that pretreatment with PPK protects the gastrointestinal tract against acute mucosal damage caused by HCl/EtOH.

Indomethacin, a nonsteroidal anti-inflammatory drug (NSAIDs), is commonly recommended in clinical practice because it reduces the inflammation by limiting the production of prostaglandins (PG) from arachidonic acids via inhibition of cyclooxygenase (COX) [23]. Changes in PG levels stimulate acid secretion, disrupting gastric homeostasis, increasing neutrophil infiltration, inducing tumor necrosis factor (TNF) expression, and disrupting the balance between free radicals and antioxidants [23]. Due to this, long-term or frequent usage of indomethacin is highly linked to a higher risk of adverse gastrointestinal events, including stomach mucosal erosion, ulceration, bleeding, and perforation [24]. PPK at all concentrations showed a significant reduction of total area of lesion in the indomethacin-induced ulcer model. However, only high concentration of PPK (50 mg/kg) significantly suppressed the microscopic gastric lesion's features. These findings showed that PG and mucus may have a role in the PPK's antiulcer action. Although the amount of PGE2 was not determined in this investigation, it is consistent with the substantial increase in mucus production observed in PPK-treated pylorus-ligated rats, which is related to the prevention of NSAID-induced stomach ulcers.

Aside from chemical-induced gastric ulcer, we further evaluate the antiulcer property of PPK by using pyloric ligation method. This is the most often utilized technique for stomach ulcer induction, since it is capable of increasing and accumulating gastric acid and pepsin production, therefore rupturing the gastrointestinal mucosal barrier [25]. In this study, PPK-treated group failed to decrease gastric juice volume, altering the pH and total acidity excluding ranitidine and the highest concentration of PPK, compared to the disease group. However, the mucus secretion from gastric wall was significantly increased by PPK treatment. We therefor suggest that PPK inhibited gastric ulcer formation in the pyloric ligation model is not likely associated with gastric acid suppression; the increased production of mucus, on the other hand, is more likely to be a mechanism behind the positive benefits of PPK.

According to a previous study, PPK includes nine of the essential amino acids out of the total of eleven [9, 26]. It also contains significant quantities of glutamic acid, glycine, and arginine [9], all of which are essential for a variety of biological processes. Tariq and Moutaery [27] reported that gastric lesion caused by stress, indomethacin, and necrotizing chemicals was dramatically reduced in mice treated with glycine. In addition, glycine also was shown to inhibit ethanol-induced depletion of nonprotein sulfhydryls and stomach wall mucus in the same study [27]. On the other hand, arginine possessed gastroprotective effect against ibuprofen-induced gastric injury in animal model [28]. Therefore, we suggest that the gastroprotective property of PPK is highly attributed by the essential amino acid, glycine, as well as arginine.

## 5. Conclusion

Taken together, the findings from this investigation clearly imply that polypeptide K (PPK) has significant antiulcerogenic effects against hydrochloride ethanol- (HCl/EtOH-), NSAIDs-, and pyloric ligation-induced gastric ulcer. This activity might be highly associated with antioxidant property of PPK which was reported previously (Ahmad et al., 2012) and active compounds, such as glycine and arginine. Therefore, PPK might be regarded as a promising source for the development of preventative and therapeutic agent for stomach ulcers. Using single-sex studies of male animals in this study was one of the limitations, as sex was a significant source of variation. The role of gender bias in preclinical research has been identified as one of the variables leading to the poor translation and replicability concerns that plague the field. Thus, it is suggested to use animals of both genders in the future studies and investigate the signaling pathway of the PPK in suppressing the gastric ulcer formation.

## Figures and Tables

**Table 1:** Antiulcerogenic effect of PPK against HCl/EtOH.

Groups	Dose (mg/kg)	Total area of lesion (mm^2^)	Inhibition (%)
HCl/EtOH only	-	44.67 ± 3.45	-
HCl/EtOH + ranitidine	100	30.33 ± 2.81^*∗*^	32.2%
HCl/EtOH + PPK	10	35.67 ± 3.84	20.1%
HCl/EtOH + PPK	25	33.16 ± 4.13	25.7%
HCl/EtOH + PPK	50	15.67 ± 2.67^*∗∗∗*^	64.9%

Statistical analysis was conducted utilizing one-way ANOVA followed by Dunnet's post hoc test (*n* = 6), and data are expressed as mean ± SEM. ^*∗*^Significantly different from HCl/EtOH group at *p* 0.05 or ^*∗∗∗*^ at *p* < 0.001.

**Table 2:** Histopathological effect of polypeptide K (PPK) on HCl/EtOH-induced gastric ulcer in rats.

Groups/features	Normal	Edema and/or vacuolation	Epithelial disruption	Erosion extending to the muscularis mucosae
HCl/EtOH only	0.67 ± 0.21	2.67 ± 0.42	5.00 ± 0.63	3.33 ± 0.67
HCl/EtOH + ranitidine (100 mg/kg)	1.50 ± 0.22 ^*∗*^	1.00 ± 0.45 ^*∗*^	1.50 ± 0.67 ^*∗*^	0.67 ± 0.67
HCl/EtOH + PPK (10 mg/kg)	0.83 ± 0.17	1.33 ± 0.42	3.50 ± 0.92	1.33 ± 0.84
HCl/EtOH + PPK (25 mg/kg)	1.50 ± 0.22 ^*∗*^	2.00 ± 0.52	2.50 ± 0.92	2.00 ± 0.89
HCl/EtOH + PPK (50 mg/kg)	1.50 ± 0.22 ^*∗*^	2.67 ± 0.42	1.50 ± 0.67 ^*∗*^	0.67 ± 0.67

Values are expressed as mean + SEM (*n* = 6). ^*∗*^*p* < 0.05 indicates significant difference against HCl/EtOH only group. HCl/EtOH: hydrochloride acid/ethanol; PPK: polypeptide K.

**Table 3:** Antiulcerogenic effect of PPK against indomethacin.

Groups	Dose (mg/kg)	Total area of lesion (mm^2^)	Inhibition (%)
Indomethacin only	-	41.33 ± 3.57	-
Indomethacin + ranitidine	100	13.67 ± 3.15 ^*∗∗∗*^	66.92%
Indomethacin + PPK	10	23.00 ± 3.38 ^*∗∗*^	44.35%
Indomethacin + PPK	25	17.33 ± 3.56 ^*∗∗∗*^	58.07%
Indomethacin + PPK	50	11.50 ± 2.01 ^*∗∗∗*^	72.18%

Data are expressed as mean ± SEM (*n* = 6). ^*∗∗*^ Significantly different from indomethacin group at *p* < 0.01 or ^*∗∗∗*^ at *p* < 0.001.

**Table 4:** Histopathological effect of polypeptide K (PPK) on indomethacin-induced gastric ulcer in rats.

Groups/features	Normal	Edema and/or vacuolation	Epithelial disruption	Erosion extending to the muscularis mucosae
Indomethacin only	0.83 ± 0.17	4.33 ± 0.84	5.00 ± 0.97	4.00 ± 1.03
Indomethacin + ranitidine (100 mg/kg)	2.67 ± 0.42 ^*∗*^	2.00 ± 0.52 ^*∗*^	2.00 ± 0.63 ^*∗*^	1.33 ± 0.84
Indomethacin + PPK (10 mg/kg)	1.00 ± 0.37	3.33 ± 0.67	4.00 ± 0.63	2.67 ± 0.84
Indomethacin + PPK (25 mg/kg)	1.67 ± 0.33	2.33 ± 0.61	2.50 ± 0.92	1.33 ± 0.84
Indomethacin + PPK (50 mg/kg)	2.00 ± 0.52	1.67 ± 0.33 ^*∗*^	1.50 ± 0.67 ^*∗*^	0.67 ± 0.67 ^*∗*^

Values are represented as the mean + SEM (*n* = 6). ^*∗*^*p* < 0.05 indicates significant difference against HCl/EtOH only group. PPK: polypeptide K.

**Table 5:** Effect of PPK on gastric juice parameters in pylorus-ligated rats.

Groups	Volume (mL)	pH (unit)	Total acidity (m equiv./L)	Ulcer area (mm2)	Protection (%)
Control	5.50 ± 0.83	3.73 ± 0.04	5847 ± 308	25.80 ± 2.79	-
Ranitidine (100 mg/kg)	3.82 ± 0.65	4.00 ± 0.26	2707 ± 330 ^*∗∗∗*^	8.75 ± 0.75 ^*∗∗∗*^	66.09%
PPK (10 mg/kg)	4.35 ± 0.88	3.07 ± 0.20	4813 ± 512	10.90 ± 1.67 ^*∗∗∗*^	57.75%
PPK (25 mg/kg)	3.50 ± 0.75	3.18 ± 0.17	4893 ± 538	8.30 ± 1.65 ^*∗∗∗*^	67.83%
PPK (50 mg/kg)	4.50 ± 0.60	3.47 ± 0.32	3926 ± 559 ^*∗*^	7.72 ± 1.69 ^*∗∗∗*^	70.08%

Results are presented as mean ± SEM (*n* = 6). ^*∗*^*p* < 0.05 and ^*∗∗∗*^*p* < 0.001 indicate significant difference compared to indomethacin only group.

**Table 6:** Effect of PPK on gastric wall mucus secretion.

Groups	Dose (mg/kg)	Gastric wall mucus (Alcian blue mg/g wet tissue)
Control	-	0.89 + 0.05
Ranitidine	100	1.39 + 0.13 ^*∗∗*^
PPK	10	1.38 + 0.15 ^*∗∗*^
PPK	25	1.73 + 0.08 ^*∗∗∗*^
PPK	50	2.60 + 0.04 ^*∗∗∗*^

Results are presented as mean ± SEM (*n* = 6). ^*∗∗*^*p* < 0.01 and ^*∗∗∗*^*p* < 0.001 indicate significant difference compared to indomethacin only group.

## Data Availability

The datasets supporting the conclusions of this study are included within the manuscript.
